# The impact of physical activity and dietary habits on glycolipemic metabolism and inflammatory markers in the elderly: a cross-sectional study

**DOI:** 10.3389/fnut.2025.1600038

**Published:** 2025-09-22

**Authors:** Wentao Zhang, Limin Zou, Jiangang Chen

**Affiliations:** ^1^Jiangxi Normal University, Nangchang, Jiangxi, China; ^2^College of Physical Education, Jinggangshan University, Ji’an, China; ^3^College of Physical Education, Beijing Normal University, Beijing, China

**Keywords:** physical activity, vitamin B, inflammatory markers, elderly, mediation analysis

## Abstract

**Objective:**

Metabolic syndrome and chronic inflammation significantly impact the quality of life of the elderly. Physical activity and dietary habits are two of the most important modifiable aspects of lifestyle. Thus, this study investigated the effects of physical activity and dietary habits on relevant biomarkers in the elderly.

**Methods:**

A total of 2349 elderly participants aged 60–75 were recruited. Physical activity was measured using the International Physical Activity Questionnaire (IPAQ) short form. Dietary habits and intakes were assessed via the Dietary Quality Questionnaire (DQQ) and 24-h recalls. Fasting blood samples were analyzed for glycolipid metabolism and inflammatory markers, such as C - reactive protein (CRP) and interleukin - 6 (IL - 6).

**Results:**

There was a close association between physical activity and diet. Moderate - intensity physical activity (MPA) was positively associated with the intake of dark green leafy vegetables (β = 0.174) and negatively associated with the intake of unprocessed red meat (β = −0.112) and deep - fried foods (β = −0.117). Both physical activity and diet affected biomarkers. Vigorous physical activity was positively correlated with high - density lipoprotein cholesterol (*r* = 0.144), while MPA was negatively correlated with blood glucose (*r* = −0.127) and CRP (*r* = −0.129). The percentage of protein intake was positively correlated with triglycerides (*r* = 0.118). Mediation analysis demonstrated the combined effects of physical activity and diet. The results showed that MPA significantly and negatively affected CRP levels, with the intake of dark green leafy vegetables mediating this relationship (*P* < 0.05). MPA also significantly and negatively affected blood glucose levels, with vitamin B12 intake mediating this relationship (*P* < 0.05).

**Conclusion:**

The study indicates that physical activity and diet interact with each other and jointly affect blood glucose and inflammation in the elderly. Diet mediates the effect of physical activity on biomarkers. Further longitudinal studies are needed to verify the findings of this study.

## 1 Introduction

The global population is aging at an unprecedented rate, making the health of older adults a critical public health priority. By 2050, the proportion of individuals aged 60 years and older is projected to rise dramatically, straining healthcare systems, economies, and social services worldwide (1). Aging is associated not only with physical and cognitive decline (2) but also with metabolic disturbances, including dysregulated glucose and lipid metabolism (3). These changes significantly impair quality of life and elevate the risk of chronic conditions such as cardiovascular disease and diabetes (4). Consequently, age-related glycolipid metabolic disorders and low-grade chronic inflammation have emerged as pressing global health challenges (5).

Aging disrupts glycolipid metabolism through mechanisms such as insulin resistance, visceral fat accumulation, and impaired lipid processing (6–8). Older adults–particularly women–exhibit reduced insulin sensitivity, exacerbated by shifts in body fat distribution and loss of skeletal muscle mass, a key site for glucose uptake (9–11). These alterations increase susceptibility to diabetes, metabolic syndrome, and cardiovascular disease, underscoring the need for interventions targeting metabolic health in aging populations.

Simultaneously, aging is characterized by chronic low-grade inflammation (“inflammaging”), marked by elevated levels of inflammatory mediators like C-reactive protein (CRP), interleukin-6 (IL-6), and tumor necrosis factor-alpha (TNF-α) (12–14). This state is further amplified in individuals with metabolic disorders (15) and contributes to atherosclerosis, bone density loss, and immune dysfunction (16, 17). Crucially, chronic inflammation and glycolipid dysregulation form a vicious cycle, accelerating age-related health decline (18).

Modifiable lifestyle factors, particularly physical activity and diet, offer promising avenues to mitigate these effects. Regular exercise improves insulin sensitivity, reduces blood glucose and lipid levels, and dampens inflammatory responses (19). Aerobic activities (e.g., walking, swimming) enhance cardiorespiratory fitness and glycemic control (20), while resistance training preserves muscle mass and metabolic rate (21). Similarly, dietary patterns influence metabolic health; high-fiber, low-glycemic diets and anti-inflammatory foods (e.g., Mediterranean diet) ameliorate dyslipidemia and inflammation (22, 23).

Numerous studies have demonstrated the independent and combined effects of physical activity and dietary interventions on metabolic and inflammatory markers in older adults. For instance, a study found that moderate-intensity exercise significantly increased postprandial carbohydrate oxidation rates after high-carbohydrate meals while reducing fat oxidation, suggesting exercise modulates nutrient metabolism in a diet-dependent manner (24). Similarly, research comparing Mediterranean and traditional Chinese diets under isocaloric conditions revealed that both dietary patterns improved glycemic control, though Mediterranean diets were more effective in reducing hypoglycemic episodes, highlighting the role of dietary composition in metabolic regulation (25).

Other investigations have explored the mechanistic pathways linking lifestyle factors to metabolic health. One study demonstrated that high-protein diets counteract post-dieting fat mass rebound by suppressing gut Lactobacillus-mediated lipid absorption, underscoring the interplay between dietary protein and gut microbiota in obesity prevention (26). Additionally, time-restricted feeding, particularly morning-based eating windows, was shown to enhance insulin sensitivity and reduce inflammation in healthy non-obese adults, suggesting meal timing as a modifiable factor in metabolic regulation (27).

Despite these advances, few studies have systematically examined how dietary habits mediate the effects of physical activity on glycolipid metabolism and inflammation in elderly populations. While some research has noted synergistic benefits–such as combined aerobic exercise and high-fiber diets improving insulin sensitivity more than either intervention alone 28–the specific mediating role of nutrients remains underexplored. This gap underscores the originality of the current study, which aims to elucidate these interactions through biomarker analysis and mediation modeling, offering novel insights for targeted lifestyle interventions in aging.

While the independent benefits of physical activity and diet are well-documented (28–30), their combined effects on glycolipid metabolism and inflammation in older adults remain underexplored. This study aims to address this gap by investigating how interactions between physical activity and dietary habits influence these outcomes. We hypothesize that: Higher physical activity levels and healthier dietary patterns will correlate with improved glycolipid profiles (e.g., lower fasting glucose, favorable lipid levels) and reduced inflammatory markers (e.g., CRP, IL-6).

This study is the first to systematically examine the synergistic effects of moderate-to-vigorous physical activity and specific dietary patterns (e.g., dark green leafy vegetable intake) on glycolipid metabolism and inflammatory markers in the elderly. It reveals the mediating role of diet–particularly dark green leafy vegetables and vitamin B12–in the relationship between physical activity and biomarkers. By providing direct evidence for combined lifestyle interventions (exercise and nutrition), this research addresses a critical gap in understanding how dietary habits mediate exercise-induced metabolic benefits. These findings offer a scientific foundation for tailored health strategies in aging populations.

## 2 Materials and methods

### 2.1 Participants

**FIGURE 1:**
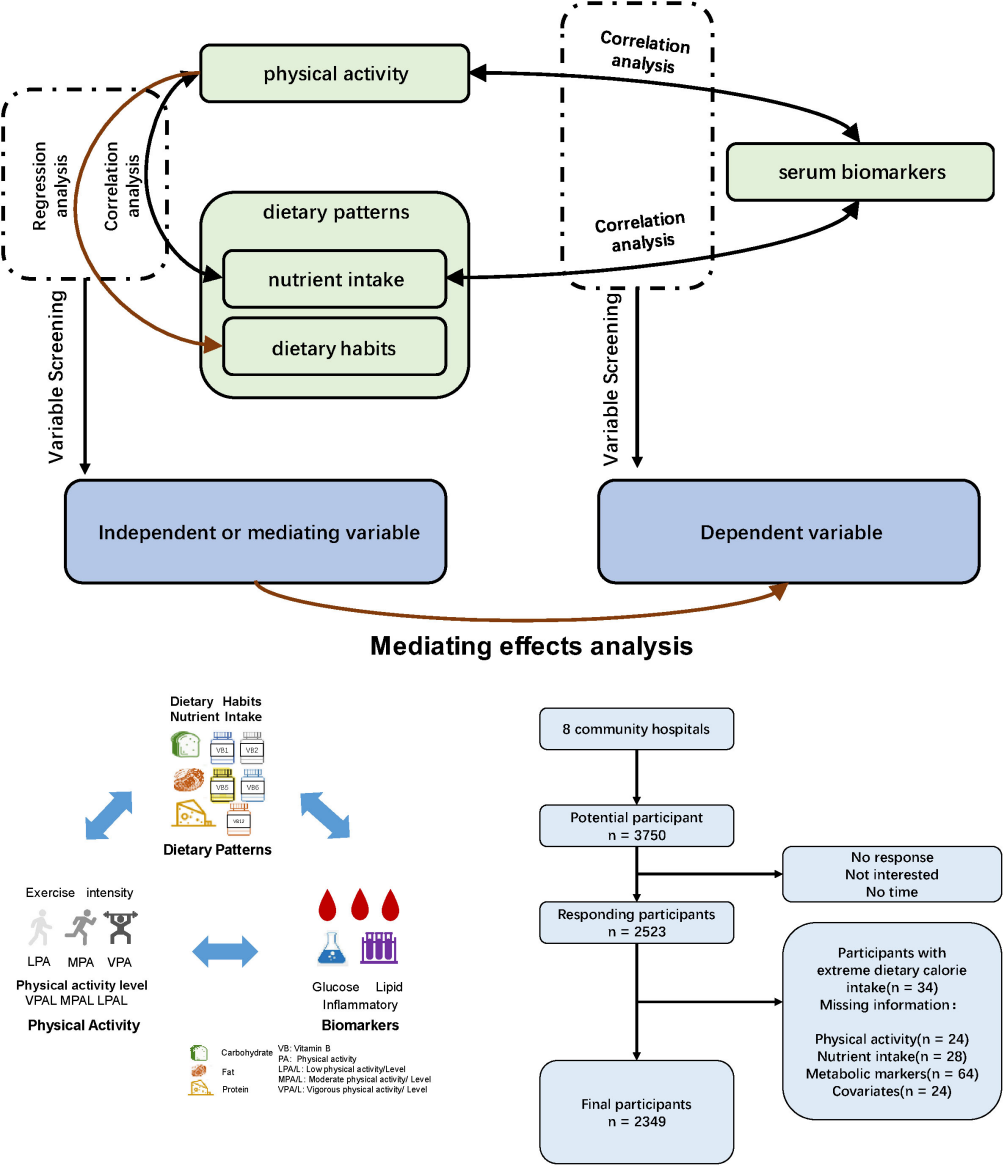
Study design flowchart.

The study was conducted in a northern city in China. Recruitment took place from September to October 2023, targeting 3750 potential participants from various community hospitals across the city. Eight communities were selected from different districts to ensure representation. Questionnaires were distributed via poster advertisements for initial screening. Out of the 2523 responses received, 34 participants with extreme dietary calorie intake and 140 who did not complete the full questionnaire were excluded. This resulted in a final sample of 2349 participants. The study design is presented in Figure 1.

Participants were included in the study if they were aged between 60 and 75 years. They had to be non-smokers and have no history of long-term alcohol consumption, defined as more than 50 g per week. Additionally, only those who were not on any medication were selected. Cognitive function was also assessed, and individuals with no cognitive impairment, as evidenced by a Montreal Cognitive Assessment (MoCA) score of 26 or higher, were included. Participants were excluded from the study if they had major acute or chronic diseases. Those self-reporting any cardiovascular or cerebrovascular diseases were also excluded. Furthermore, individuals using weight-loss medications or with a history of depression, Parkinson’s disease, Alzheimer’s disease, severe mental disorders, stroke, or brain injury were not eligible for the study.

The study protocol was approved by the Ethics Committee of Jiangxi Normal University (RB_0343_56). The research was conducted in accordance with the ethical standards of the 1964 Helsinki Declaration.

### 2.2 Physical activity assessment

Physical activity levels of participants were quantified using the International Physical Activity Questionnaire (IPAQ) short form, a seven-item instrument that evaluates the frequency and duration of physical activities across different intensities, including vigorous (VPA), moderate (MPA), and low (LPA) physical activity. Administered by trained personnel through one-on-one interviews with elderly participants, the IPAQ short form ensures comprehension and clarity of responses, and is recognized for its high reliability and validity in the Chinese elderly population (31). Trained personnel received training through a combination of online and offline sessions. The training covered the structure and scientific basis of the IPAQ, standardized instructions for administering the questionnaire, and how to explain items clearly to participants. Standardized scripts were developed to maintain consistency. Based on total Metabolic Equivalent of Task (MET) minutes per week, participants were categorized into three activity levels: Low Physical Activity level (LPAL) for scores below 600 MET⋅min⋅wk^–1^, Moderate Physical Activity level (MPAL) for scores between 600 and 3000 MET⋅min⋅wk^–1^, and Vigorous Physical Activity level (VPAL) for scores of 3000 MET⋅min⋅wk^–1^ or higher.

### 2.3 Dietary data collection

Dietary intake data were collected using the Dietary Quality Questionnaire(DQQ), which comprises yes/no questions regarding the consumption of sentinel foods corresponding to 24 food groups over the past 24 h. The Chinese version of the DQQ has been validated for its ability to capture food group consumption patterns within the Chinese population (32).

#### 2.3.1 Nutrient intake assessment

To assess dietary habits and estimate daily intakes of carbohydrates, fats, proteins, and vitamin B complex from the diet, three 24-h dietary recalls were conducted by trained dietitians over two weekdays and one weekend day. These recalls were collected via validated telephone interviews. The dietitians received structured training to ensure standardized and accurate data collection. This included comprehensive guidance on the 24-h dietary recall method, practice in interviewing techniques (e.g., rapid listing, probing for forgotten foods, estimating portion sizes), and calibration using visual aids. They were also trained to use neutral questioning techniques to minimize bias. Continuous quality control was maintained through supervised mock interviews and calibration meetings.

Participants were first educated on the proper use of visual aids for portion size estimation of common foods. This training featured real plates, glasses, and food models, complemented by hands-on experiential communication to enhance understanding and accuracy.

Food consumption data were collected through multiple 24-h dietary recall interviews, ensuring a comprehensive capture of dietary intake patterns. Macronutrient and micronutrient intakes were calculated using the nutritional software MetaDieta (Meteda s.r.l., Ascoli-Piceno).

Daily total food intake was expressed in grams per day (g/day), with vitamin B1-6 intake reported in milligrams per day (mg/day), vitamin B9-12 reported in micrograms per day (μg/day). Energy intake values falling outside the range of 1000–6000 kcal/day were considered implausible and were excluded from the analysis.

### 2.4 Measurement of glycolipemic metabolism and inflammatory markers

#### 2.4.1 Blood sample collection

Fasting blood samples were collected in the early morning by nurses at local community hospitals. Participants were instructed to fast for at least 8 h prior to the blood draw to ensure the accuracy of the metabolic measurements.

#### 2.4.2 Metabolic markers

Blood glucose, glycated hemoglobin (HbA1c), and lipid profile, including triglycerides, total cholesterol, high-density lipoprotein (HDL), and low-density lipoprotein (LDL), were measured by the community hospitals and reported accordingly.

#### 2.4.3 Inflammatory markers

C-reactive protein (CRP), a widely recognized marker of systemic inflammation, was also assessed by the community hospitals and reported accordingly. The concentration of IL-6, a key cytokine involved in regulating immune responses and inflammation, was measured using the Human IL-6 ELISA Kit (YEASENELISA). The assay was performed strictly according to the manufacturer’s instructions, with a detection range of 0.16–10.5 pg/mL. The analyses were conducted in the university laboratory. Blood samples were immediately placed in insulated boxes with ice after collection. The samples were transported to the laboratory within 10 min and centrifuged at 1500 rpm for 10 min to separate plasma/serum for analysis. The measurements were conducted using a microplate reader. If analysis could not be conducted immediately, samples were stored at −80 °C until analysis, with strict limits on freeze-thaw cycles (≤1). The overall inter-assay coefficient of variation for IL-6 was 6.7%.

### 2.5 Covariates

In the analysis, potential confounding variables were controlled for to isolate the effects of physical activity and dietary habits on metabolic outcomes. These covariates encompassed socio-demographic factors such as gender (male or female), age (ranging from 60 to 75 years), marital status (married, widowed, divorced, or single), educational attainment (classified into primary school or below, junior high school, high school, and higher education), and annual household income. Additionally, lifestyle factors were considered, with sleep quality being evaluated using the Pittsburgh Sleep Quality Index (PSQI), a self-rated questionnaire that assesses sleep quality and disturbances over a 1-month period, yielding a score from 0 to 21, where higher scores denote poorer sleep quality (33).

### 2.6 Statistical analysis

Data analysis was conducted using IBM SPSS version 26 (IBM Corp., Armonk, NY, USA). To assess the normality of the distribution, the Shapiro-Wilk test was applied. Data that deviated from a normal distribution were reported as medians with interquartile ranges (IQR). The Mann-Whitney U test was employed to evaluate significant intergroup differences in physical activity and nutrient intake between genders. Comparisons of rate type data were made using the chi-square test. Spearman’s rank correlation coefficient was utilized to assess the strength and direction of correlations between variables. For the categorical variables derived from the Dietary Quality Questionnaire (DQQ), multiple linear regression analyses were conducted to explore the associations with other variables. To investigate the mediating relationships among physical activity, diet, and various biomarkers, we employed the PROCESS macro for SPSS (34). This tool enabled us to test the mediating effects of physical activity and dietary habits on the relationships between biomarkers. We alternately considered physical activity and diet as independent or mediating variables, with each biomarker serving as the dependent variable. This approach facilitated the identification of significant mediating relationships. Statistical significance was set at *P* < 0.05.

## 3 Results

### 3.1 Sociodemographic characteristics

Table 1 presents the sociodemographic characteristics stratified by gender. Comparative analyses revealed statistically significant differences between males and females in terms of education and income (*P* = 0.017). Males demonstrated significantly higher levels of total physical activity compared to females (*P* = 0.038). Differences in other indices were not statistically significant.

**TABLE 1:** Comparison of basic demographic characteristics between the genders.

Vriables	Male (*n* = 1057)	Female (*n* = 1292)	*P*
Age	60.00 (4.00)	60.00 (4.00)	0.356
BMI (kg/m^2^)	22.55 (4.14)	22.70 (4.60)	0.735
Education			0.017
<6 years	5.6%	16.5%	/
6–9 years	22.2%	26.9%	/
9–12 years	25.7%	34.9%	/
12–16 years	27.1%	17.7%	/
>16 years	19.4%	3.6%	/
Family income (monthly)			0.017
<500 yuan	13.9%	24.9%	/
500–1500 yuan	14.6%	13.3%	/
1500–2500 yuan	10.4%	11.2%	/
2500–3500 yuan	20.1%	24.1%	/
>3500 yuan	41.0%	26.5%	/
PA (MET⋅min/wk)	940.00 (1970.00)	650 (1530.00)	0.038
Glucose (mmol/L)	4.96 (0.56)	5.01 (0.46)	0.179
HbA1c (%)	6.47 (0.39)	6.50 (0.32)	0.179
Triglycerides (mmol/L)	1.04 (0.57)	1.06 (0.54)	0.546
Total cholesterol (mmol/L)	4.85 (0.92)	4.83 (0.90)	0.964
HDL cholesterol (mmol/L)	1.57 (0.36)	1.61 (0.36)	0.893
LDL cholesterol (mmol/L)	2.78 (0.80)	2.79 (0.74)	0.702
CRP (mg/L)	2.41 (1.74)	2.49 (2.12)	0.190
IL-6 (pg/mL)	2.74 (1.29)	2.77 (1.11)	0.421

Values are presented as medians with interquartile ranges (IQR) for continuous variables and as percentage (%) for categorical variables. Continuous variables were compared using Mann-Whitney U test. Categorical variables were compared using the Chi-square test. *P*-values denote the significance level for differences between males and females. BMI, body mass index; PA, physical activity; HbA1c, glycated hemoglobin; HDL, high-density lipoprotein; LDL, low-density lipoprotein; CRP, C-reactive protein; IL-6, interleukin-6.

### 3.2 Correlation analysis

Table 2 results show that vigorous-intensity physical activity was positively correlated with the percentage of protein intake from total caloric intake, vitamins B5, B6, and B12 (*r* = 0.121; *r* = 0.128; *r* = 0.129), and negatively correlated with the percentage of caloric intake from carbohydrates (*r* = −0.085). Moderate-intensity physical activity was positively correlated with vitamin B12 (*r* = 0.104).

**TABLE 2:** Correlation between physical activity and nutrient intake.

	TPA	VPA	MPA	LPA
Vitamin B1	−0.098	0.005	−0.026	−0.118
Vitamin B2	−0.021	0.022	0.060	−0.091
Vitamin B3	−0.078	0.016	−0.017	−0.130
Vitamin B5	−0.053	0.121*	0.018	−0.111
Vitamin B6	−0.058	0.128*	−0.031	−0.110
Vitamin B9	−0.005	0.058	0.035	−0.040
Vitamin B12	−0.054	0.001	0.104*	−0.106
%TCI-CHO	−0.056	−0.085*	0.017	−0.050
%TCI-PRO	0.084	0.129*	0.044	0.106
%TCI-FAT	−0.065	0.009	−0.054	−0.087

VPA, vigorous - intensity physical activity; MPA, moderate - intensity physical activity; LPA: low - intensity physical activity; TPA: total physical activity.

*Indicates statistical significant.

Table 3 results, derived from multivariate linear regression controlling for confounding factors, indicate a positive association between moderate-intensity physical activity and the intake of dark green leafy vegetables (β = 3.201). Conversely, moderate-intensity physical activity was negatively associated with the intake of unprocessed red meat (β = −1.963) and deep-fried foods (β = −2.393).

**TABLE 3:** Significant associations between physical activity and dietary habits.

Dependent variable	Independent variable	β	*t*	*P*
Dark green leafy vegetables	VPA	−0.056	−1.112	0.267
	MPA	0.174	3.201	0.001
	LPA	0.002	0.030	0.976
Unprocessed red meat	VPA	−0.045	−0.853	0.394
	MPA	−0.112	−1.963	0.049
	LPA	0.056	1.015	0.311
Deep fried foods	VPA	−0.001	−0.031	0.975
	MPA	−0.117	−2.393	0.017
	LPA	0.020	0.426	0.670

VPA, vigorous - intensity physical activity; MPA, moderate - intensity physical activity; LPA, low - intensity physical activity.

Table 4 indicates that vigorous levels of physical activity were significantly positively correlated with high-density lipoprotein cholesterol (HDL-C) (*r* = 0.144). Moderate levels of physical activity were significantly negatively correlated with blood glucose (*r* = −0.127) and C-reactive protein (CRP) (*r* = −0.129). Additionally, the percentage of protein intake relative to total caloric intake was significantly positively correlated with triglycerides (*r* = 0.118).

**TABLE 4:** Correlation of physical activity, nutrient intake and serum biomarkers.

	GLU	HbA1c	TG	TC	HDL	LDL	CRP	IL-6
PA	0.033	0.033	0.050	0.062	−0.009	0.053	0.003	0.038
VPA	−0.055	−0.055	0.082	0.082	0.144*	0.055	0.036	0.058
MPA	−0.127*	0.027	0.116	0.073	−0.041	0.080	−0.129*	0.048
LPA	0.036	0.036	0.006	0.043	0.024	0.022	0.012	0.013
Vitamin B1	−0.006	−0.006	−0.067	−0.032	0.053	−0.069	−0.002	0.026
Vitamin B2	0.059	0.059	0.045	0.057	0.019	0.051	0.043	−0.018
Vitamin B3	0.046	0.046	0.049	0.026	0.056	−0.008	−0.008	−0.024
Vitamin B5	0.045	0.045	0.059	0.043	0.018	0.032	0.015	−0.031
Vitamin B6	0.052	0.052	0.036	0.015	0.032	−0.006	−0.012	0.000
Vitamin B9	0.009	0.009	−0.031	−0.033	0.005	−0.069	−0.006	−0.007
Vitamin B12	0.074	0.074	0.027	0.029	0.088	0.023	0.059	−0.037
%TCI-CHO	0.011	0.011	−0.053	−0.060	0.026	−0.074	−0.026	−0.008
%TCI-PRO	0.082	0.082	0.118*	0.061	−0.010	0.060	0.016	−0.029
%TCI-FAT	−0.010	−0.010	0.084	0.037	−0.036	0.046	0.041	0.018

VPA, vigorous - intensity physical activity; MPA, moderate - intensity physical activity; LPA, low - intensity physical activity; PA, physical activity; HbA1c, glycated hemoglobin; HDL, high-density lipoprotein; LDL, low-density lipoprotein; TG, triglycerides; TC, total cholesterol; CRP, C-reactive protein; IL-6, interleukin-6; TCI, total calorie intake; CHO, carbohydrates; PRO, protein.

*Indicates statistical significant.

### 3.3 Mediating effect analysis

**FIGURE 2:**
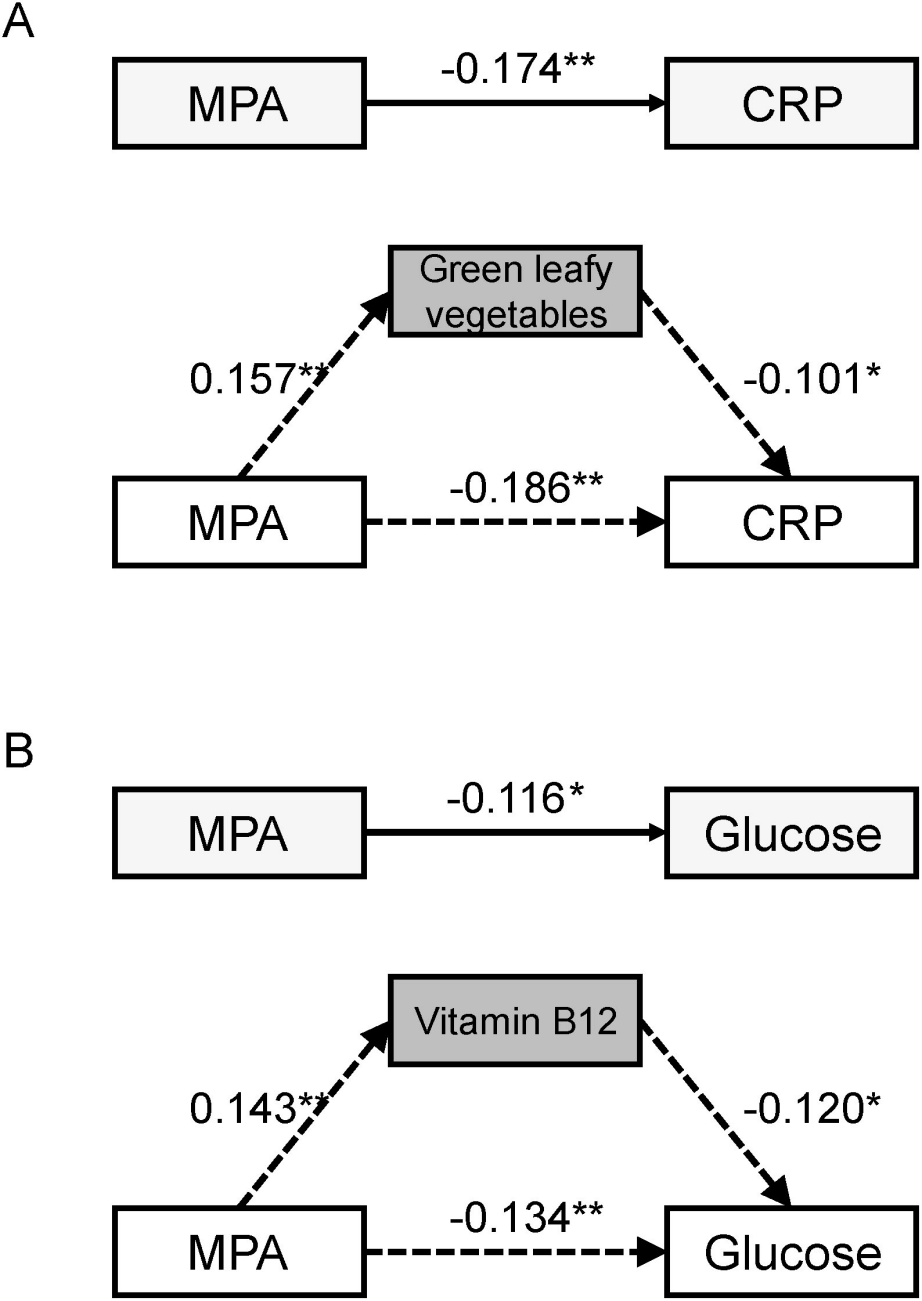
The mediation model of diet mediating the effects of physical activity on biomarkers. **(A)** Significant mediating effects between MPA, dietary habits and CRP. **(B)** Significant mediating effects between MPA, nutrient intake and glucose. MPA, moderate - intensity physical activity; CRP, C - reactive protein. *P < 0.05, **P < 0.01.

Table 5 results demonstrate that, after controlling for confounding factors, moderate-intensity physical activity has a significant effect on C-reactive protein levels, with the intake of dark green leafy vegetables mediating this relationship, indicating a significant mediating effect. Furthermore, after controlling for confounding factors, moderate-intensity physical activity also significantly affects blood glucose levels, with vitamin B12 intake mediating this relationship, indicating a significant mediating effect Figure 2.

**TABLE 5:** Significant mediating effects between physical activity, dietary habits and serum biomarkers.

Independent variable	Dependent variable	β	*t*	*P*
1 MPA	CRP	−0.174	−3.458	0.001
2 MPA	Dark green leafy vegetables	0.157	3.281	0.001
3 MPA	CRP	−0.186	−3.670	0.000
Dark green leafy vegetables		−0.101	−2.017	0.044
1 MPA	Glucose	−0.116	−2.290	0.023
2 MPA	Vitamin B12	0.143	2.843	0.005
3 MPA	Glucose	−0.134	−2.615	0.009
Vitamin B12		−0.120	−2.354	0.019

MPA, moderate - intensity physical activity; CRP, C - reactive protein.

## 4 Discussion

### 4.1 Analysis of correlation results

#### 4.1.1 Correlation analysis of physical activity, nutrient intake and serum biomarkers

This study aimed to examine how physical activity and dietary habits collectively influence glycolipid metabolism and inflammatory markers in elderly individuals. Our findings demonstrate three key outcomes: high-intensity physical activity significantly improves HDL-C levels, moderate-intensity exercise effectively reduces blood glucose and CRP levels, and dietary protein proportion shows a complex relationship with triglyceride levels.

The results reveal that higher intensity physical activity was positively correlated with HDL-C levels. Previous studies have shown that regular high-intensity exercise enhances lipid metabolism by increasing HDL-C concentrations, which may substantially lower atherosclerosis risk (35). Furthermore, research indicates that exercise promotes cardiovascular health through optimized lipid metabolism and decreased visceral fat accumulation (36).

Regarding glucose regulation and inflammation control, moderate-intensity physical activity exhibited significant negative associations with both blood glucose and CRP levels. These observations align with studies demonstrating that moderate exercise improves insulin sensitivity while simultaneously reducing systemic inflammation (37). This dual benefit suggests moderate physical activity may serve as an effective intervention for metabolic disorders in aging populations.

Nutritional analyses uncovered important relationships between dietary patterns and metabolic markers. The positive association between protein intake proportion and triglyceride levels warrants particular attention. While studies confirm that high-protein diets can enhance fat oxidation, research also indicates that excessive protein intake may adversely affect lipid profiles (38). Additionally, the complex interactions observed between macronutrient ratios and metabolic regulation confirm that dietary composition plays a critical role in elderly health outcomes (39).

These findings collectively emphasize the synergistic importance of physical activity and dietary habits in maintaining metabolic health. High-intensity exercise proves particularly beneficial for lipid profiles, while moderate exercise shows advantages for glucose metabolism and inflammation control. Simultaneously, balanced dietary patterns emerge as equally crucial factors. For elderly populations, comprehensive lifestyle interventions incorporating appropriate exercise regimens and nutritional strategies may offer optimal protection against metabolic disorders and support healthy aging.

#### 4.1.2 Correlation analysis between physical activity and nutrient intake

This study aimed to investigate the relationship between physical activity levels and nutritional intake patterns in elderly individuals. The key findings demonstrate that: high-intensity physical activity was positively associated with protein and B vitamin intake while negatively correlated with carbohydrate proportion; moderate-intensity exercise showed a specific positive correlation with vitamin B12 intake.

The results revealed significant positive correlations between high-intensity physical activity and the intake of protein, vitamins B5, B6, and B12. Previous research has demonstrated that high-intensity exercise stimulates greater protein consumption and increases requirements for B vitamins involved in protein metabolism and energy production (40). Studies indicate these nutrients play vital roles in muscle recovery and metabolic processes following intense physical activity (41).

Conversely, high-intensity exercise showed a negative association with carbohydrate intake proportion. Research suggests this may reflect altered energy substrate utilization during high-intensity activity, where the body preferentially utilizes fats and proteins over carbohydrates as primary fuel sources (42). These findings highlight how exercise intensity influences macronutrient selection and metabolic demands.

Regarding moderate-intensity activity, the positive correlation with vitamin B12 intake is particularly noteworthy. Vitamin B12 is known to be essential for neurological function and energy metabolism. Studies have shown that moderate physical activity may increase physiological demands for this vitamin, potentially explaining the observed dietary pattern (43).

These findings collectively suggest that different exercise intensities are associated with distinct nutritional intake patterns in elderly individuals. High-intensity activity appears linked to increased protein and B vitamin consumption with reduced carbohydrate proportion, while moderate exercise shows specific associations with vitamin B12 intake. These exercise-nutrient relationships may have important implications for optimizing dietary recommendations to support physical activity in aging populations.

The interaction between physical activity and nutritional intake observed in this study provides valuable insights for developing targeted lifestyle interventions. Future research should explore whether these associations reflect conscious dietary choices or physiological adaptations to different exercise regimens.

#### 4.1.3 Significant associations between physical activity and dietary habits

This study aimed to examine the relationship between moderate-intensity physical activity (MPA) and dietary patterns in elderly individuals. The key findings demonstrate that: MPA was positively associated with consumption of dark green leafy vegetables, while showing negative associations with intake of unprocessed red meat and fried foods.

The positive correlation between MPA and dark green leafy vegetable intake suggests that regular moderate exercise may promote healthier food choices. Research has shown that dark green leafy vegetables are rich sources of vitamin K, folate, fiber and antioxidants, whose consumption can enhance immune function and lower chronic disease risk (44). These findings support the notion that physical activity may influence dietary preferences toward more nutrient-dense options.

Conversely, the negative associations between MPA and consumption of unprocessed red meat and fried foods indicate that active individuals may naturally reduce their intake of less healthy food options. Studies have demonstrated that regular physical activity tends to decrease consumption of high-fat, high-cholesterol foods like red meat, which are linked to increased cardiovascular and metabolic disease risk (45). Similarly, research indicates that active individuals typically consume fewer fried foods, which are high in trans fats and calories associated with obesity and heart disease (46).

These findings collectively suggest that moderate-intensity physical activity may promote healthier dietary patterns through multiple mechanisms. First, it appears to increase preference for nutrient-rich vegetables. Second, it seems to decrease consumption of potentially harmful food items. The combined effect of these dietary shifts could significantly contribute to better long-term health outcomes in elderly populations.

The observed associations between physical activity and dietary habits provide important insights for developing integrated lifestyle interventions. Future research should investigate whether these relationships reflect conscious behavior changes or more subtle physiological and psychological adaptations to regular exercise. Additionally, longitudinal studies could help determine if these dietary changes mediate the known health benefits of physical activity.

### 4.2 Significant mediating effects analysis between physical activity, dietary habits and serum biomarkers

This study aimed to investigate the mediating role of dietary habits in the relationship between moderate-intensity physical activity (MPA) and key serum biomarkers in elderly individuals. The findings demonstrate that: MPA showed significant negative associations with CRP and blood glucose levels while positively correlating with vitamin B12 levels, with dietary habits (particularly dark green leafy vegetable intake) serving as important mediators of these relationships.

The observed negative association between MPA and CRP levels aligns with existing research showing that physical activity can modulate inflammatory responses. Studies have demonstrated that regular moderate exercise reduces CRP concentrations, potentially lowering cardiovascular disease risk through anti-inflammatory mechanisms (47). CRP elevation, a well-established marker of chronic inflammation, is consistently reduced through physical activity interventions (48).

The mediating role of dark green leafy vegetable intake in the MPA-CRP relationship represents a key finding. Research indicates that physical activity promotes healthier food choices, particularly increasing consumption of nutrient-dense vegetables (49). These vegetables provide antioxidants and anti-inflammatory compounds that may synergize with exercise to further reduce systemic inflammation.

Regarding glucose metabolism, MPA showed both direct and indirect (diet-mediated) effects on blood glucose regulation. Previous studies confirm that moderate exercise enhances insulin sensitivity through multiple pathways (50). Our results extend this understanding by suggesting that dietary improvements, particularly increased vegetable intake, may amplify these metabolic benefits.

The positive association between MPA and vitamin B12 levels may reflect exercise-induced changes in dietary patterns. As vitamin B12 plays crucial roles in neurological function and erythropoiesis, research suggests that physically active individuals tend to consume more B12-rich foods, potentially explaining this relationship (51).

These findings have important clinical implications. The dual approach of combining moderate physical activity with dietary modifications (particularly increased vegetable consumption) appears particularly effective for improving metabolic and inflammatory markers in elderly populations. This synergistic effect may offer enhanced protection against chronic diseases compared to either intervention alone (52).

Future research should explore whether these mediating relationships vary by demographic factors or baseline health status. Additionally, intervention studies could help determine optimal combinations of physical activity and dietary changes for maximal health benefits in aging populations.

### 4.3 Limitations

This study has several limitations. The cross-sectional design prevents conclusions about causality. Self-reported physical activity and dietary data may be affected by recall bias. The sample was limited to elderly individuals in specific communities, which may affect generalizability. Future research using longitudinal designs and objective measurements would help confirm these findings.

## 5 Conclusion

Physical activity levels and dietary habits in older adults play an important role in metabolic health. Specifically, moderate-intensity physical activity was significantly associated with high-density lipoprotein cholesterol levels, whereas lower levels of physical activity were negatively associated with blood glucose and C-reactive protein (CRP) levels. In addition, dietary protein intake was significantly and positively correlated with triglycerides. These results suggest that by promoting moderate physical activity and optimizing dietary structure, glycolipid metabolism and reducing inflammation levels in older adults can be effectively improved. Therefore, health interventions for older adults should emphasize the integrated management of physical activity and nutritional intake to enhance their overall metabolic health.

## Data Availability

The original contributions presented in this study are included in this article/supplementary material, further inquiries can be directed to the corresponding author.
